# Vancomycin-induced immune hemolytic anemia: a case report and literature review

**DOI:** 10.3389/fimmu.2026.1842837

**Published:** 2026-09-17

**Authors:** Ruochen Zhang, Yanan Wang, Zhongying Wang, Yu Tao, Sujuan Zheng, Shenghao Xu, Ling Wang, Dong Xiang, Jiang Wu

**Affiliations:** 1 Department of Blood Transfusion, Ren Ji Hospital, School of Medicine, Shanghai Jiao Tong University, Shanghai, China; 2 Blood Group Reference Laboratory, Shanghai Institute of Blood Transfusion, Shanghai Blood Center, Shanghai, China

**Keywords:** diagnosis and treatment of hemolysis, drug antibody titers, drug-induced immune hemolytic anemia (DIIHA), serological test, vancomycin

## Abstract

Drug-induced immune hemolytic anemia (DIIHA) is a rare but potentially life-threatening adverse drug reaction. Although more than 150 drugs have been reported to induce DIIHA, vancomycin, a glycopeptide antibiotic widely used in clinical practice, is rarely associated with hemolysis, making it prone to underdiagnosis or misdiagnosis. This article reports a case of vancomycin-induced DIIHA in a 64-year-old female patient after right revision knee arthroplasty (RKA). This study investigated the serological tests associated with vancomycin and tracks drug antibody titers through longitudinal monitoring during clinical diagnosis and treatment. Additionally, a literature review identified and summarized five previously reported cases of vancomycin-induced immune hemolytic anemia. Clinicians should maintain a high index of suspicion for hemolysis associated with vancomycin, including its use in bone cement. Early recognition, prompt withdrawal of the offending agent, and transfusion support are imperative for improving patient outcomes with DIIHA.

## Introduction

DIIHA represents a clinically significant and potentially reversible form of hemolytic anemia (HA). Although rare, it is a life-threatening adverse drug reaction triggered by immune activation, which elicits antibodies directed against autologous red blood cells (RBCs) and subsequent hemolysis (1). Clinical features include fever, pain, hemoglobinuria, dyspnea, and hypotension. Severe cases may progress to coma, posing a life-threatening risk (2).

The clinical presentation of DIIHA closely resembles that of primary autoimmune hemolytic anemia and other hemolytic disorders, rendering differential diagnosis challenging (3). Thus, DIIHA is frequently underrecognized, resulting in delayed diagnosis and treatment. Prompt recognition and withdrawal of the offending drug are critical for optimizing patient outcomes, and any suspected drug should be discontinued immediately upon clinical suspicion.

With the widespread use of novel therapeutic agents, the incidence of DIIHA has been increasing (4, 5), underscoring the need for heightened clinical vigilance and proactive risk assessment. Vancomycin remains a cornerstone for treating severe Gram-positive bacterial infections, particularly those caused by methicillin-resistant *Staphylococcus aureus* (MRSA) and methicillin-resistant *Staphylococcus epidermidis* (MRSE) (6). However, vancomycin has also been associated with a range of adverse effects, including thrombocytopenia (7, 8), drug reaction with eosinophilia and systemic symptoms (DRESS) (9),and DIIHA (10, 11). These findings have raised clinical concerns regarding the safety profile of vancomycin and highlight the necessity for individualized diagnostic and therapeutic strategies, as well as tailored transfusion management.

This study monitored drug antibody dynamics and the clinical course in a patient with vancomycin-induced DIIHA, and reviewed previously reported cases for comparative analysis of salient features, thereby offering practical insights for clinical management.

## Case description

### Patient information and clinical data

A 64-year-old woman was admitted for right RKA. Preoperative testing determined her blood type as B/CcDEe. Antibody screening was negative via saline method but positive on gel card. The surgery proceeded smoothly without the need for blood transfusion. Postoperatively, she received prophylactic antimicrobial therapy with vancomycin and voriconazole (12), and her initial recovery was uneventful.

On postoperative day (POD) 7, she developed acute respiratory distress accompanied by generalized petechiae, ecchymoses, and elevated serum lactate. After pulmonary embolism was excluded, she was transfer to the ICU and started on empiric antimicrobial therapy with vancomycin and piperacillin-tazobactam. By POD 8, the patient progressed to an acute hemolytic crisis, manifested by a progressive decline in hemoglobin, hemoglobinuria, elevated total bilirubin (predominantly indirect), increased lactate dehydrogenase, and decreased haptoglobin. Peripheral blood analysis showed a reticulocyte count >7% and a B-lymphocyte proportion of 29.92%. Both antibody screening and direct antiglobulin test (DAT) were strongly positive.

The patient was a married, parous woman with a history of multiple pregnancies and deliveries, and no prior exposure to blood transfusions, solid organ transplantation, or hematopoietic stem cell transplantation. Laboratory evaluations, including viral serologies, autoantibody screening, serum protein electrophoresis with immunofixation, peripheral blood flow cytometry (to exclude B-cell lymphoproliferative disorders such as chronic lymphocytic leukemia), and serum tumor biomarker assays, were all unremarkable (13). After excluding viral infection, autoimmune disease, thrombotic microangiopathy, paraproteinemia, lymphoproliferative disorders, and chemical injury, drug-induced hemolysis was suspected (14), since several studies have reported the associations between drug exposures and immune HA (15). Serological testing demonstrated a positive antibody screen by the saline method alongside a pan-reactive result on the gel card. All potentially offending drugs were consequently discontinued, and an emergency transfusion was requested. Prior to transfusion, the patient and her family were thoroughly informed of the potential risks, particularly hemolytic transfusion reactions, and written informed consent was obtained.

### Serological identification of drug-dependent antibodies

#### Identification of drug-dependent antibodies

To identify the drug responsible for the patient’s hemolysis, drug-dependent antibodies against vancomycin, voriconazole, and piperacillin were detected using the immune-complex method (**Figure 1A**). Each drug was prepared at a concentration of 1 mg/mL and incubated with the patient’s serum (16). Positive agglutination was observed only in the presence of vancomycin, while results for the other agents were negative. Further validation was performed using vancomycin from multiple sources, lots, and formulations to exclude the influence of excipients or impurities (16), and all of these preparations induced positive agglutination. Furthermore, the patient’s RBC eluate also demonstrated agglutination upon reaction with vancomycin. These findings confirm the presence of anti-vancomycin antibodies in the patient, which likely played a key role in mediating immune-mediated hemolysis.

**Figure 1:**
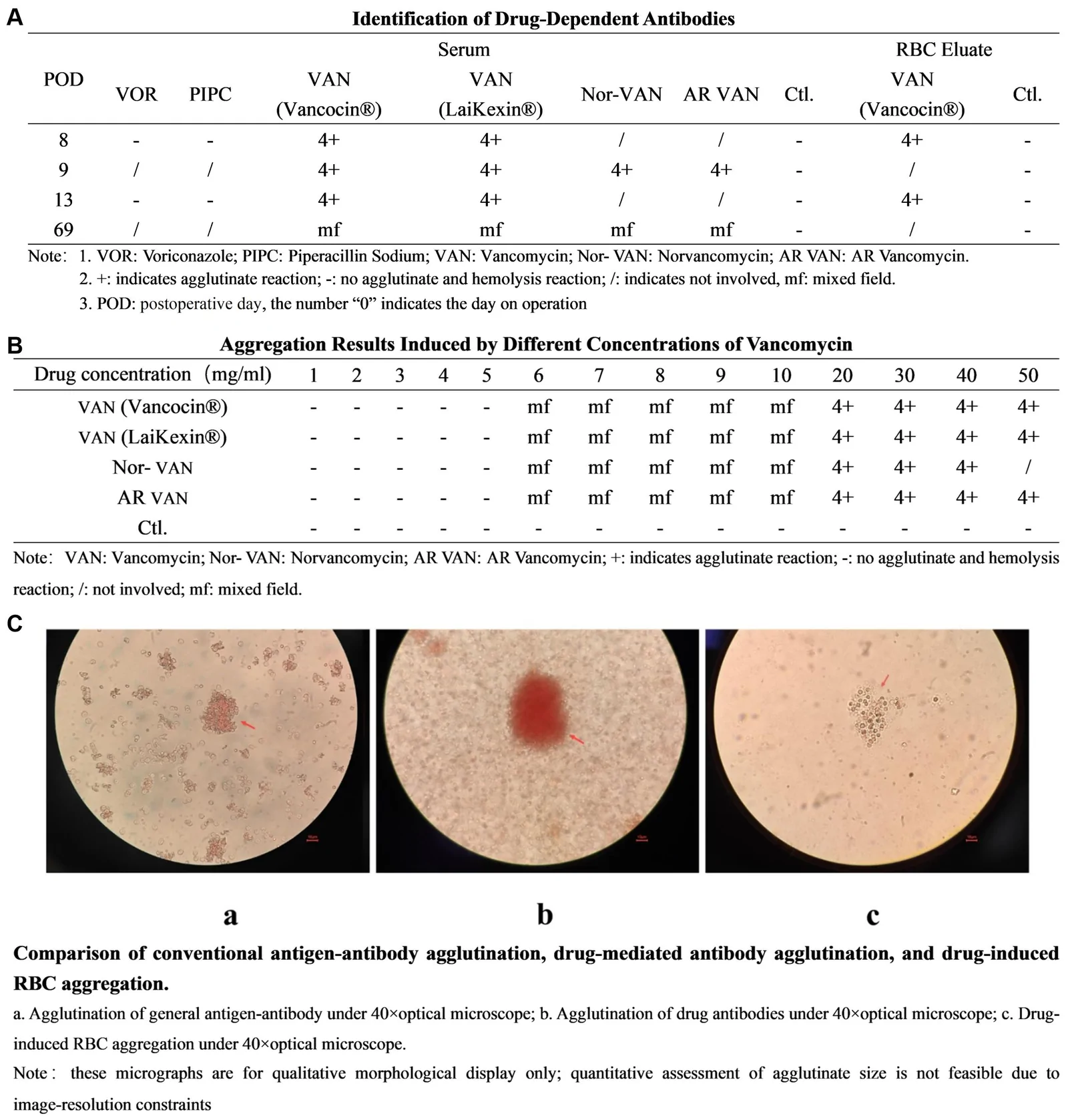
Serological identification of drug-dependent antibodies. **(A)** Identification of drug-dependent antibodies; **(B)** Aggregation results of standard O group cells induced by different concentrations of Vancomycin; **(C)** Comparison of conventional antigen-antibody agglutination, drug-mediated antibody agglutination, and drug-induced RBC aggregation.

#### Vancomycin induced RBCs aggregation

At high concentrations, the protonated amino groups of vancomycin can neutralize the negative charges of sialic acid residues on the erythrocyte membrane surface, thereby reducing electrostatic repulsion and inducing reversible linear rouleaux formation (17). In this study, serial dilutions of vancomycin were prepared at concentrations of 1, 2, 3, 4, 5, 6, 7, 8, 9, 10, 20, 30, 40, and 50 mg/mL. These solutions were incubated with a 1% suspension of group O indicator RBC at 37 °C for 15 min, followed by centrifugation and agglutination grading (**Figure 1B**). The results showed that RBC aggregation occurred at vancomycin concentrations >5 mg/mL, and a 4+ aggregation intensity was reached at concentrations >10 mg/mL.

In this study, the morphological features of RBC aggregation induced by 10 mg/mL vancomycin were examined under microscopy and compared with those of classical antigen-antibody immune reactions and drug-dependent antibody-mediated immune agglutination (**Figure 1C**). Microscopic examination revealed distinct morphological differences among the three types of agglutination. Blood group antigen-antibody complexes formed dense, compact clumps, drug-induced immune antigen-antibody complexes appeared moderately loose, and vancomycin-induced spontaneous RBC agglutination displayed a scattered, loosely stacked pattern. Saline replacement and blank control assays further confirmed that high-concentration vancomycin could indeed trigger RBC aggregation. (Only key microscopic findings are presented in the main text; the detailed reproducible serological protocol is provided in the **Supplementary Materials**).

Vancomycin administration both before and during surgery adhered strictly to the package insert. Moreover, serum drug concentration levels were significantly lower than those required to induce spontaneous erythrocyte agglutination *in vitro*. Therefore, it can be inferred that the hemolytic reaction observed in this patient was mediated by a drug-induced immune antigen-antibody reaction, rather than RBC agglutination.

### Identification of drug antibody subtypes and titer monitoring

#### Subtype identification of vancomycin-dependent antibodies

In the absence of vancomycin, antibody screening using the saline tube method was negative, whereas the gel card method detected anti-Fy^b^ alloantibodies.

Upon vancomycin addition, the saline method revealed anti-N antibodies, and the gel card demonstrated a pan-reactive pattern. Genotyping confirmed the patient’s MN blood group as M-N^+^. These findings indicate that the vancomycin-dependent antibodies comprised both IgG and IgM isotypes, with the IgM component exhibiting anti-N specificity, the IgG component lacked apparent specificity.

#### Subsequent longitudinal monitoring of antibody titers

On POD 8, the titer of vancomycin-dependent IgG antibodies was 1:64, which gradually decreased to 1:2 by POD 111. On POD 9, the titer of vancomycin-dependent IgM antibodies was 1:1024, declining to 1:4 by POD 111. Following drug discontinuation, the serum vancomycin concentration reached its peak on POD 8 (3.56 μg/mL) and declined thereafter; vancomycin was undetectable on POD 13. By POD 123, both antibodies were serologically undetectable (**Figure 2**).

**Figure 2:**
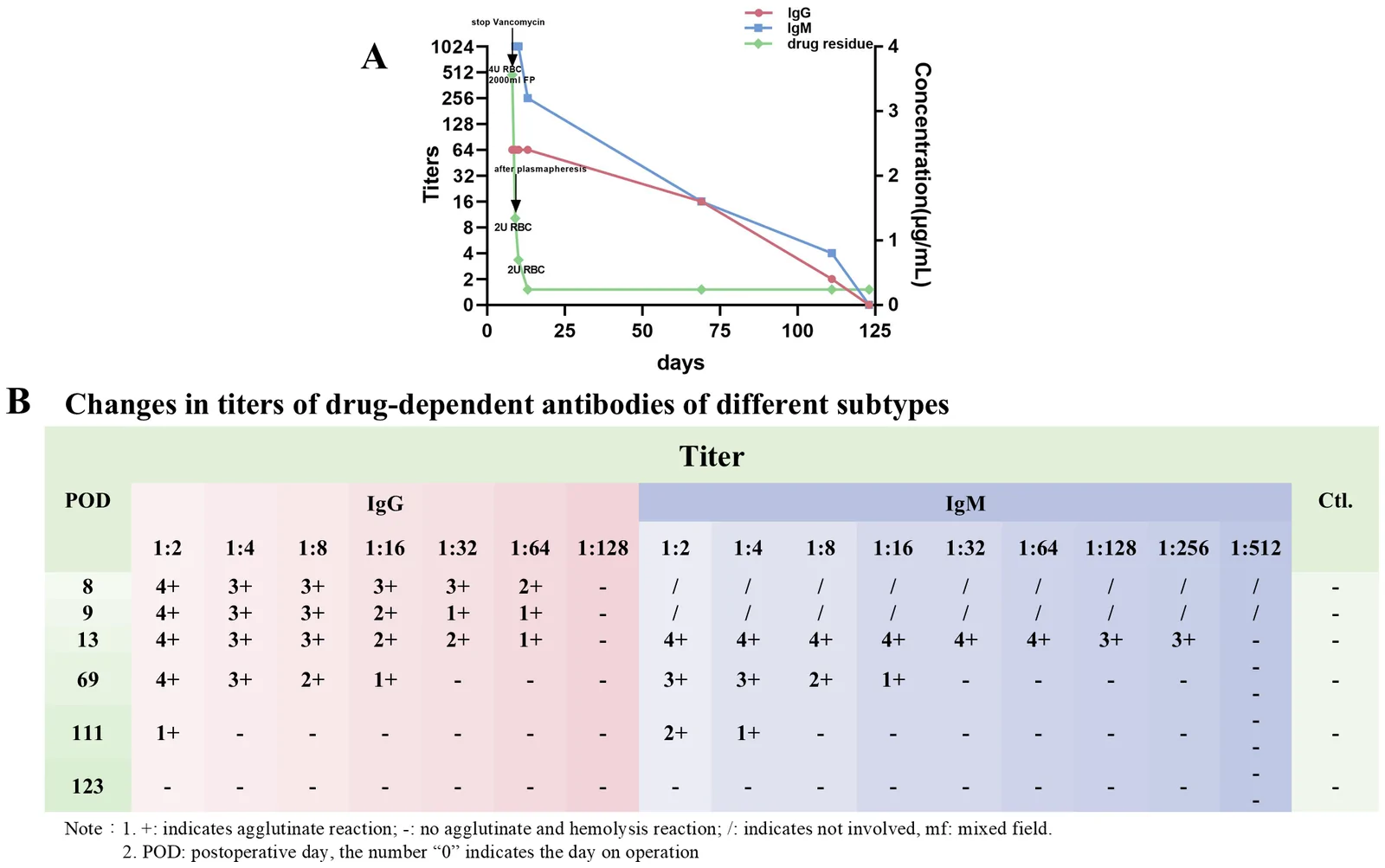
Dynamic changes in vancomycin antibody titers and drug concentration.

### Diagnostic assessment and therapeutic intervention

Upon confirmation of vancomycin-induced hemolysis, vancomycin was discontinued immediately, and therapeutic plasma exchange was urgently initiated (18), with infusion of 2000 mL of frozen plasma. Aggressive transfusion support was also administered, consisting of 4 units of crossmatched RBCs on POD 8, and 2 units each on POD 9 and POD 10. Concomitant treatment included corticosteroid therapy (19) combined with intravenous immunoglobulin (IVIG) By POD 13, serum vancomycin concentration was undetectable and the anti-C3 antibody had become negative. Abnormal laboratory parameters reflecting cardiac, hepatic, and renal function, as well as coagulation profiles, gradually normalized (**Figure 3**). The patient was transferred from the ICU on POD 24 and was discharged on POD 28.

**Figure 3:**
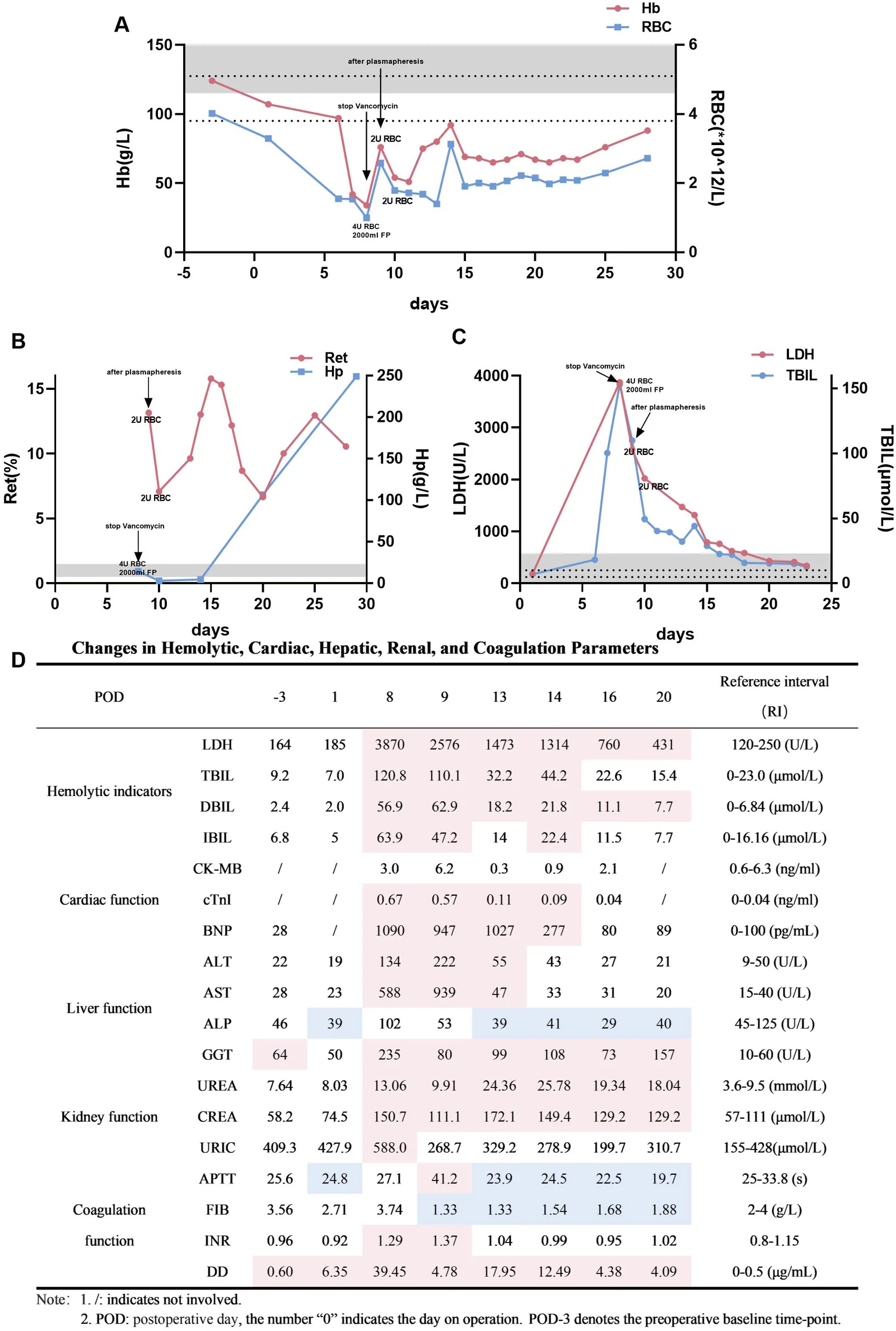
Changes in hemolytic, cardiac, hepatic, renal, and coagulation parameters. **(A)** Changes in hemoglobin and RBCs levels; **(B)** Changes in reticulocyte and haptoglobin levels; **(C)** Changes in lactate dehydrogenase and direct bilirubin levels; **(D)** Changes in hemolytic, cardiac, hepatic, renal, and coagulation parameters.

## Discussion

DIIHA is an acquired hemolytic anemia resulting from antibody-mediated accelerated destruction of RBCs induced by drugs. Severe cases can be life-threatening, warranting urgent etiological diagnosis (2). The underlying mechanisms are primarily classified as drug-dependent, drug-independent, and non-immunological. Although vancomycin-induced DIIHA is rare, the increasing number of relevant case reports, driven by its widespread use in the treatment of postoperative orthopedic infections and MRSA infections, warrants clinical vigilance.

Consistent with previously reported cases (10, 11, 16, 20, 21), we describe a case of severe DIIHA attributed to vancomycin. This underscores the importance of suspecting DIIHA in the event of an unexpected decline in hemoglobin levels during antibiotic therapy. A systematic literature review was conducted in accordance with the PRISMA-S guidelines for systematic reviews of case reports. Ultimately, five eligible cases were identified and included in the qualitative analysis. A comparative analysis between our patient and those described in previous reports is summarized in **Table 1**, revealing consistent findings across cases.

**Table 1:** Comparative analysis of Vancomycin-induced immune hemolytic anemia cases.

Author (year)	Age/sex	Clinical diagnosis	Route of exposure	Latency to hemolysis	Nadir Hb and time-point of nadir	Blood product support	Details of vancomycin dose in bone cement	Case follow-up duration	DAT changes	Antibody specificity	Key outcome
Current case	64/F	Right RKA	IV: 1.0 g q12h	POD 7	34 g/L, POD 8	8 U washed RBCs + therapeutic plasma exchange (2000ml)	VAN-containing cement; exact dose unreported	123 d (from POD 8 to POD 131)	Peak: IgG(2+), C3(3+); POD13: C3(-); 123 d: DAT(-)	VAN-dependent antibody (IgG) with N antigen specificity (IgM)	Hb stabilized after VAN discontinuation; discharged at day 28
Gniadek et al. (2018) (16)	61/F	Infection after right RKA	IV + bone cement (1 g)	POD 7	50 g/L, POD 7	5 U RBCs	Spacer loaded with VAN 1 g	51 d (from POD 7 to POD 58)	POD7: IgG(-), C3(1+); POD8: IgG(1+), C3(3+); POD51: IgG(-), C3(1+)	VAN-dependent antibody (IgG)	Hemolysis resolved after VAN discontinuation
Sudhakaran et al. (2019) (11)	72/M	Infection after THA	IV + bone cement (6 g)	14 days after initiation	57 g/L, Day 14	Hemodialysis	Spacer loaded with VAN 6 g	> 90 d (follow-up extended to 3 months post-discharge)	Serial DAT: NR	Not tested (clinical diagnosis)	Hemolysis persisted after IV discontinuation; resolved after spacer removal
Siddiqui et al. (2023) (10)	75/M	Infection after right RKA	IV	3 days after initiation	54 g/L, Day 3	Multiple RBC transfusions	No cement/spacer used	> 21 d	Onset: IgG(+); serial follow-up DAT: NR	Not tested (clinical diagnosis)	Hb began to rise 3 days after VAN cessation
Song et al. (2025) (20)	58/M	Infection, MRSA bacteremia	IV	6 days after re-initiation	42 g/L, Day 6 post-re-challenge	2 U RBCs + IVIG	No cement/spacer used	> 30 d (follow-up until hospital discharge)	Onset: DAT positive; serial strength: NR	VAN-dependent antibody (confirmed by reference laboratory)	Hb stabilized after VAN discontinuation; discharged at day 30
Xie et al. (2025) (21)	50/M	Left RKA	IV: 1.0 g q12h + bone cement	POD 8	44 g/L, POD 8	3 U N^-^ RBCs	VAN-mixed cement; exact dose unreported	1 d (from hemolysis onset to death)	Peak: IgG(2+), C3(4+); no post-event follow-up	VAN-dependent antibody with N antigen specificity	Died of cerebral infarction 1 day after VAN discontinuation

RKA, revision knee arthroplasty; THA, total hip arthroplasty; M, man. F, female; DAT, direct antiglobulin test; Hb, hemoglobin; IV, intravenous; q12h, every 12 hours; POD, postoperative day; RBCs, red blood cells; IVIG, intravenous immunoglobulin; U, units; VAN, vancomycin.

Vancomycin-induced DIIHA typically manifests within 3 to 14 days after initiation of intravenous therapy. Our patient developed a severe hemolytic reaction on POD 7. Moreover, re-exposure following initial sensitization may precipitate a more rapid and severe hemolytic response. In this case, the patient had previously received vancomycin for infection prophylaxis during removal of the right knee prosthesis and placement of a cement spacer 9 months earlier, which likely induced the initial production of anti-vancomycin antibodies (IgM/IgG). Antibody titers subsequently declined to undetectable levels over the following months. Re-exposure to vancomycin during current RKA may have induced a rapid anamnestic B cell response, resulting in substantial antibody production. These antibodies formed immune complexes with the drug, which attached to RBC surfaces, activated the complement system, and ultimately induced severe acute intravascular hemolysis (22). The clinical presentation was characterized by classic signs and symptoms of acute hemolysis, including a precipitous drop in hemoglobin to a critical level (3.4-5.7 g/dL), elevated indirect bilirubin and lactate dehydrogenase (LDH), undetectable haptoglobin, reticulocytosis, and hemoglobinuria. In severe instances, fever, tachycardia, dyspnea, hemodynamic instability, and even multiorgan failure and shock may occur-findings consistent with the clinical course observed in our patient.

Meanwhile, the patient exhibited a marked elevation in lymphocyte count during the hemolytic episode, with a predominance of B lymphocytes, suggesting humoral immune activation. However, this finding alone cannot establish the diagnosis of DIIHA. The diagnosis in our case was made by comprehensive assessment integrating DAT results, *in-vitro* drug-dependent antibody assays, and the temporal relationship between vancomycin administration and hemolysis onset.

In addition, DAT in DIIHA is typically positive, with a characteristic dual positivity for IgG and C3d, reflecting complement activation secondary to immune complex-mediated hemolysis. Antibody screening tests, which are negative prior to drug exposure, become positive following the onset of hemolysis. In this context, detection of drug-dependent antibodies constitutes the diagnostic gold standard. Serological testing was performed using both the drug coating method and the immune complex method. While high concentrations of vancomycin can induce spontaneous RBC aggregation, vancomycin does not form covalent bonds with the RBC membrane. Therefore, detection of anti-vancomycin antibodies must rely on the immune complex method, in which soluble vancomycin is added to the patient’s serum together with indicator RBCs, and agglutination is assessed.

In contrast to previous reports in which the implicated antibodies were predominantly IgG, the vancomycin-dependent antibodies identified in our case comprised both IgM and IgG isotypes, with the IgM component exhibited anti-N specificity. Genotyping confirmed the patient’s MN blood type as M^-^N^+^, suggesting that the anti-N antibody is likely drug-dependent. This observation is consistent with previous reports (16, 21). Both Xie et al. and Gniadek et al. have identified vancomycin-dependent antibodies with clear anti-N specificity. These findings imply that antibody production may be influenced by specific protein or glycoconjugate structures on the RBC membrane, providing further support for the concept that drug-dependent antibodies can exhibit targeted specificity for blood group antigens. Similarly, piperacillin-dependent antibodies have been reported to show e antigen specificity (23), though the underlying mechanisms remain unclear and warrant further investigation.

The patient also harbored an independent anti-Fy^b^ IgG alloantibody, which was not vancomycin-dependent. As an irregular alloantibody within the Duffy blood-group system, anti-Fy^b^ is most commonly induced by sensitization through pregnancy or transfusion. It can cause incompatible cross-matching; transfusion of Fy^b^-positive RBCs can trigger delayed hemolytic transfusion reactions and may also cause hemolytic disease of the fetus and newborn. This alloantibody may coexist with drug-dependent antibodies. In patients with DIIHA, it does not directly mediate drug-related hemolysis but may exacerbate RBC destruction. Once anti-Fy^b^ is identified, Fy^b^-negative RBCs should be selected for transfusion regardless of whether drug-dependent antibodies are concurrently detected. Preoperatively, the patient was clinically stable. She reported no history of blood transfusion but had multiple pregnancies and deliveries, with no history of organ or hematopoietic stem cell transplantation and no prior episodes of hemolysis. This anti-Fy^b^ antibody most likely originated from previous pregnancies or other immunologic stimuli. Since anti-Fy^b^ can only be produced by Fy^b^-negative individuals, the patient was presumed to be Fy^b^-negative. However, due to the unavailability of stored specimens, retrospective Duffy phenotyping and genotyping could not be performed. Therefore, the Fy^b^-negative status was inferred solely from antibody identification results.

We also longitudinally monitored IgM and IgG antibody titers from the onset of hemolysis through convalescence. The IgM component, which exhibited anti-N specificity, reached an initial titer as high as 1:1024, while the IgG titer peaked at 1:64. The synergistic effects of these two antibody classes likely contributed to the induction and exacerbation of acute intravascular hemolysis and hemoglobinuria, necessitating immediate drug discontinuation, therapeutic plasma exchange, and supportive transfusion. Serum drug concentration was monitored simultaneously to assess its metabolic profile. Vancomycin was substantially cleared from the patient’s circulation by POD 13. By POD 123, both antibodies were serologically undetectable, and the patient was successfully discharged. This suggests that, with timely intervention, the clearance of drug-dependent antibodies *in vivo* occurs over approximately four months. To our knowledge, this is the first report to characterize the progressive decline in vancomycin-dependent antibody titers over time. accompanied by longitudinal follow-up of relevant clinical and organ function parameters. Based on the pathogenic mechanism of DIIHA, hemolysis will not be exacerbated when only drug-dependent antibodies persist *in vivo* in the absence of the causative drug. This is consistent with the clinical recovery observed in our patient after vancomycin was cleared from circulation. Dynamic titer monitoring mainly characterizes the *in-vivo* clearance kinetics of drug-dependent antibodies, provides evidence for subsequent immunological research, and may offer a potential safe time-window for future re-exposure to the offending drug.

Through this case report of a unique presentation of vancomycin-associated DIIHA, we comprehensively analyze recently published cases, exploring the clinical manifestations, serological features, potential pathogenic mechanisms, and treatment strategies associated with vancomycin-induced DIIHA. Accumulating evidence suggests that vancomycin primarily causes intravascular or extravascular hemolysis through the formation of drug-dependent antibodies-most commonly of the IgG isotype but occasionally in combination with IgM. Notably, these antibodies may demonstrate preferential binding to specific blood group antigens, such as the N antigen.

Clinicians should remain vigilant regarding the risk of persistent hemolysis associated with vancomycin use. For vancomycin-impregnated bone cement or spacers, prophylactic dosing for infection prevention ranges from 0.25 to 1 g of vancomycin per 40 g of bone cement, whereas for the treatment of periprosthetic joint infection, doses of 2–4 g per 40 g are commonly used, with a maximum of up to 5–8 g. A substantial burst release of the drug occurs within 48 hours after implantation, followed by a rapid decline (24). The *in vitro* antibacterial activity persists for a maximum of 21 to 35 days. *In vivo*, vancomycin may be continuously eluted at low concentrations for weeks to months, as it becomes encapsulated by tissue fluid, fibrin, and biofilm, thereby sustaining low serum drug levels over an extended period (25). Notably, published orthopedic case reports have demonstrated that in previously sensitized patients, persistent micro-elution of vancomycin from bone cement can maintain ongoing DIIHA-related hemolysis even after intravenous vancomycin has been discontinued. In some such cases, surgical removal of the spacer is required to resolve the hemolysis.

For patients receiving vancomycin-whether via intravenous administration or implantation of vancomycin-impregnated bone cement or spacers-baseline laboratory testing should be obtained prior to treatment, and hemoglobin levels should be closely monitored during the 3 to 14 day high-risk period following initiation. Vancomycin-induced DIIHA should be strongly suspected in patients with recent vancomycin exposure who present with an unexplained rapid decline in hemoglobin accompanied by hemoglobinuria or jaundice, along with laboratory evidence of hemolysis and a newly positive direct antiglobulin test (DAT), frequently dual-positive for IgG and C3, after other potential etiologies have been excluded. Vancomycin must be discontinued immediately and replaced with an alternative non-cross-reactive antibiotic. A comprehensive serological workup, including DAT, elution studies, irregular antibody identification, and detection of soluble vancomycin-dependent antibodies, should be completed. Transfusion support is indicated for life-threatening anemia. If blood group-specific antibodies, such as anti-N, are identified, RBC units negative for the corresponding antigen should be selected for transfusion. Early recognition, prompt withdrawal of the offending antibiotic, and implementation of therapeutic plasma exchange together with supportive transfusion are essential for effective management of DIIHA.

This patient developed life-threatening fulminant vancomycin-induced DIIHA and subsequently received multiple intensive rescue interventions. Therapeutic plasma exchange was promptly initiated to rapidly remove circulating pathogenic antibodies and immune complexes, while fresh-frozen plasma was administered to replenish consumed coagulation factors (18, 26). Methylprednisolone sodium succinate was given intravenously as follows: a 40 mg bolus and 100 mg infusion on POD 7 and 8; 80 mg once daily from POD 9 to 22; followed by a switch to oral prednisone acetate at 50 mg twice daily from POD 23 to 29 (19, 26).Adjunctive therapy also included IVIG at a total dose of 20 g. In addition, aggressive transfusion support was provided: 4 units of cross-matched RBCs were transfused on POD 8, and 2 units on each of POD 9 and 10, to maintain adequate tissue oxygenation and provide a therapeutic window for other interventions.

Such comprehensive intensive interventions are indicated only for fulminant hemolysis with a high risk of mortality, as observed in this case. Most mild cases of DIIHA resolve with drug discontinuation and supportive care alone. High-quality randomized evidence supporting the routine use of corticosteroids or IVIG in DIIHA is lacking; both agents represent empirical rescue measures. Immunomodulatory agents should be tapered gradually once hemolysis is adequately controlled, to avoid overtreatment and reduce both adverse effects and economic burden.

This study has several limitations. First, no stored specimens were available for retrospective Duffy blood group phenotyping or genotyping, which prevented in-depth characterization of the anti-Fy^b^ alloantibody. Second, standardized quantitative agglutination assays and third-party slide review were not performed, reflecting inherent constraints of retrospective serological investigations. Third, his study mainly focused on vancomycin-induced DIIHA. Regarding piperacillin associated drug- dependent antibodies, we did not further explore the interaction *in vivo* with the e antigen after preliminary serological exclusion testing for drug-dependent antibodies. Fourth, multiple interventions including drug discontinuation, high dose corticosteroids, plasma exchange, and transfusion were implemented concurrently during the hemolytic crisis; consequently, the therapeutic effect cannot be attributed to any single modality.

## Data Availability

The raw data supporting the conclusions of this article will be made available by the authors, without undue reservation.
