# Supplementary material for: Vancomycin-induced immune hemolytic anemia: a case report and literature review
Source: Front Immunol. 2026 Sep 17;17:1842837. doi: 10.3389/fimmu.2026.1842837 (PMC13627317; doi:10.3389/fimmu.2026.1842837)
Supplement: Supplementary file 1 [file Supplementaryfile1.pdf]

Supplementary Materials

Serological Examination

The saline method or microcolumn agglutination method was used to perform ABO, Rh, and Kidd blood group antigen detection, direct antiglobulin test (DAT), antibody screening and identification, cross-matching test, absorption-elution test, and polyethylene glycol (PEG) enhancement test. All methods were performed strictly in accordance with the serological examination procedures specified in the *Association for the Advancement of Blood* & *Biotherapies* (AABB) or the reagent instructions.

Table 1 Results of Specific Antibody Screening

VIAL	Rh	Rh-hr						Kell				Duffy		Kidd		Lewis		P	MNS				Luth.	Colt.	Xg	RESULTs			
		D	C	E	c	e	C^W	K	k	Kp^a	Js^a	Fy^a	Fy^b	Jk^a	Jk^b	Le^a	Le^b	P_1	M	N	S	s	Lu^a	Co^b	Xg^a	POD0		POD8	
																										Saline	Gel	Saline	Gel
I	CCDee R_1R_1	+	+	-	-	+	/	-	+	/	/	+	+	+	-	-	+	+	+	+	-	+	/	/	/	-	+	+	+
II	ccDEE R_2R_2	+	-	+	+	-	/	-	+	/	/	+	-	-	+	-	+	-	+	-	+	-	/	/	/	-	-	-	+
III	ccddee rr	+	-	+	+	+	/	-	+	/	/	+	+	+	-	+	-	-	+	+	-	+	/	/	/	-	+	+	+

Note: +: indicates agglutinate reaction; -: no agglutinate and hemolysis reaction; /: indicates not involved.

Table 2 Results of DAT Subtyping

POD	Self	DAT		
		Anti-IgG	Anti-C3d	Ctl.
0	/	/	/	/
8	2+	2+	2+	-
9	2+	2+	3+	-
10	2+	2+	1+	-
13	-	2+	-	-
14	-	2+	-	-

16	-	-	-	-

Note: 1. +: indicates agglutinate reaction; -: no agglutinate and hemolysis reaction; /: indicates not involved.
2. POD: postoperative day, the number “0” indicates the day on operation.

Detection of Drug-Dependent Antibodies

Immune complex method

A 1 mg/mL drug solution was prepared. 50 µL of 1% standard group O RBC suspension and 50 µL of patient serum were added to microcolumn gel well; the negative control well 50 µL of AB pooled plasma was substituted for patient serum. Then 50 µL of the corresponding drug solution was added to each well. Following incubation at 37 °C for 15 min, the card was centrifuged and the results were recorded.

Drug coating method

A 5 mg/mL drug solution was prepared and then incubated with an equal volume of a 3–5% standard group O RBC suspension at 37 °C for 1 h. Washed cells were resuspended as 1% coated RBCs. For testing, 50 µL coated RBCs were loaded into microcolumn gel well; uncoated RBCs served as negative control. Subsequently, 50 µL patient serum was added to each well; an additional control used AB pooled plasma. Following incubation at 37 °C for 15 min, the card was centrifuged and the results were recorded.

Drug-dependent antibody screening

To identify the drug responsible for the patient’s hemolysis, drug-dependent antibodies against vancomycin, voriconazole, and piperacillin were detected using the immune-complex method. Each drug was prepared at a concentration of 1 mg/mL and incubated with the patient’s serum. Normal pooled AB serum was used simultaneously as a negative control. Positive agglutination was observed only in the presence of vancomycin, while results for the other agents were negative. Further validation was performed using vancomycin from multiple sources, lots, and formulations to exclude the influence of excipients or impurities, and all of these preparations induced positive agglutination. Furthermore, the patient’s RBC eluate also demonstrated agglutination upon reaction with vancomycin. These findings confirm the presence of anti-vancomycin antibodies in the patient, which likely played a key role in mediating immune-mediated hemolysis.

Table 3 Identification of Drug Antibody Components in the Patient

Time (Days)	serum							RBC Eluate	
	Voriconazole	Piperacillin Sodium	Vancomycin (Vancocin)	Vancomycin (LaiKexin)	Norvancomycin	AR Vancomycin	Ctl.	Vancomycin (Vancocin)	Ctl.

8	-	-	4+	4+	/	/	-	4+	-
9	/	/	4+	4+	4+	4+	-	/	-
13	-	-	4+	4+	/	/	-	4+	-
69	/	/	mf	mf	mf	mf	-	/	-

Note: 1. +: indicates agglutinate reaction; -: no agglutinate and hemolysis reaction; /: indicates not involved, mf: mixed field.

2. The numbers in "Days" column indicate the day after the surgery, the number "0" indicates the day on surgery.

Drug Aggregation Test

Drug solutions were prepared at concentrations of 1, 2, 3, 4, 5, 6, 7, 8, 9, 10, 20, 30, 40, and 50 mg/mL and then mixed with an equal volume of a 1% standard group O RBC suspension respectively. Following incubation at 37 °C for 15 min, the card was centrifuged and the results were recorded.

Table 4 Aggregation Results Induced by Different Concentrations of Vancomycin

Drug concentration（mg/ml)	1	2	3	4	5	6	7	8	9	10	20	30	40	50
VAN (Vancocin®)	-	-	-	-	-	mf	mf	mf	mf	mf	4+	4+	4+	4+
VAN (LaiKexin®)	-	-	-	-	-	mf	mf	mf	mf	mf	4+	4+	4+	4+
Nor- VAN	-	-	-	-	-	mf	mf	mf	mf	mf	4+	4+	4+	/
AR VAN	-	-	-	-	-	mf	mf	mf	mf	mf	4+	4+	4+	4+
Ctl.	-	-	-	-	-	-	-	-	-	-	-	-	-	-

Note: VAN: Vancomycin; Nor- VAN: Norvancomycin; AR VAN: AR Vancomycin; +: indicates agglutinate reaction; -: no agglutinate and hemolysis reaction; /: not involved; mf: mixed field.

Comparison of Different Agglutination Patterns

Antigen-antibody agglutination

Patient group-B red blood cells were washed three times and diluted to prepare a 3-5% RBC suspension. One drop of the red-cell suspension and one drop of anti-B antibody were added into a test tube, followed by centrifugation. The results were observed and interpreted under a microscope.

Drug-mediated antibody agglutination

A 1 mg/mL drug solution was prepared. 50 μL of 1% standard group O RBC suspension and 50 μL of patient serum were added to a test tube, followed by 50 μL of the prepared drug solution. The mixture was incubated at 37 °C for 15 min, then centrifuged, and the results were observed under a microscope.

Drug-induced RBC aggregation

The results showed that RBC aggregation occurred at vancomycin concentrations >5 mg/mL, and a 4+ aggregation intensity was reached at concentrations >10 mg/mL. Drug solutions were prepared at concentrations of 10 mg/mL and then mixed with an equal volume of a 3-5% standard group O RBC suspension respectively in a test tube. Following incubation at 37 °C for 15 min, the results were observed under a microscope.

Saline replacement confirmation test

The supernatant of the reaction tube was discarded, and an equal volume of normal saline was added to resuspend red blood cells, followed by centrifugation. Complete dispersion of agglutinates indicated rouleaux formation, whereas persistent agglutination suggested true immune-mediated agglutination. After saline addition, agglutination caused by general antigen-antibody reactions and agglutination of drug-dependent antibodies remained present, while drug-induced RBC aggregation disappeared. Meanwhile, the drug in the drug-induced RBC-aggregation group was replaced with normal saline, and no agglutination was observed under the microscope.

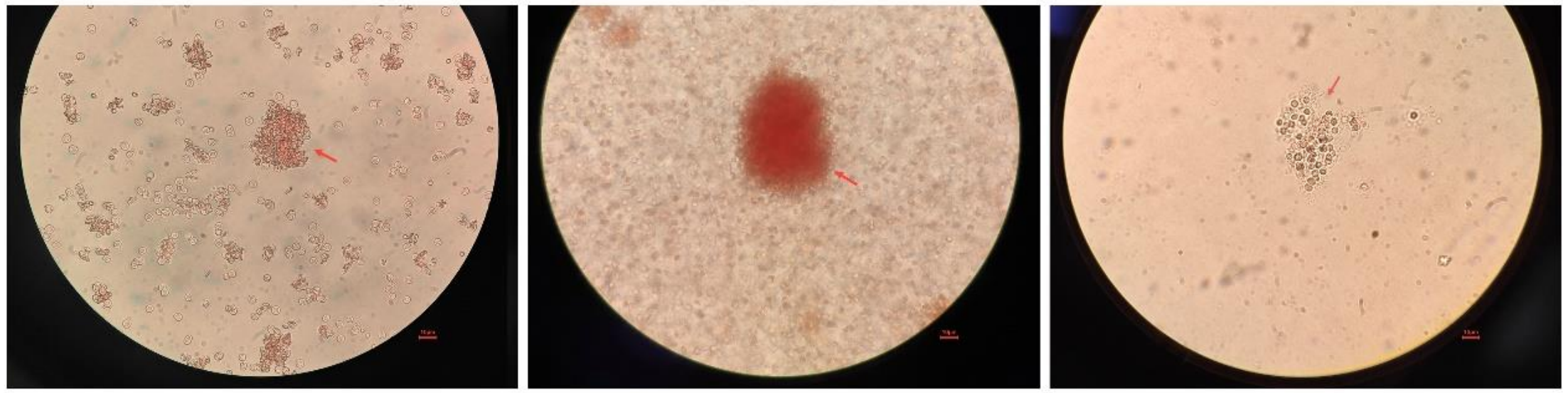

A B C

Fig 1. Comparison of conventional antigen-antibody agglutination, drug-mediated antibody agglutination, and drug-induced RBC aggregation.

A. Agglutination of general antigen-antibody under 40×optical microscope; B. Agglutination of drug antibodies under 40×optical microscope; C. Drug-induced RBC aggregation under 40×optical microscope.

Identification of Drug-Dependent Antibodies

Antibody identification was performed by reacting patient serum with panel cells. The tube method was used for identification of IgM-type antibodies. 50 μL of panel cells and 50 μL of patient serum were added to test tubes, followed by addition of 50 μL PBS or 50 μL vancomycin solution (1 mg/mL). The mixtures were centrifuged, and results were observed.

The microcolumn gel-card method was applied for identification of IgG-type antibodies. 50 μL of panel cells and 50 μL of patient serum were dispensed into microcolumn gel-card wells. 50 μL PBS or 50 μL vancomycin solution (1 mg/mL) was added respectively. After incubation at 37 °C for 15 min, the gel-cards were centrifuged and the results were read. In the absence of vancomycin, antibody screening using the saline tube method was negative, whereas the gel card method detected anti-Fy^b alloantibodies. Upon vancomycin addition, the saline method revealed anti-N antibodies, and the gel card demonstrated a pan-reactive pattern. Genotyping confirmed the patient's MN blood group as M^-N^+. These findings indicate that the vancomycin-dependent antibodies comprised both IgG and IgM isotypes, with the IgM component exhibiting anti-N specificity, the IgG component lacked apparent specificity.

Table 5 Results of Anti-Vancomycin Antibodies Typing

N0.	Rh-Hr	Rh-Hr						Kell						Duffy		Kidd		Lewis		P	MNS				Luther		Xg	RESULT			
																												Saline		Gel	
		C	D	E	c	e	C^w	K	k	Kp^a	Kp^b	Js^a	Js^b	Fy^a	Fy^b	Jk^a	Jk^b	Le^a	Le^b	P_1	M	N	S	s	Lu^a	Lu^b	Xg^a	+PBS	+ vancomycin	+PBS	+ vancomycin
1	$R_1^wR_1$	+	+	-	-	+	+	-	+	+	+	-	+	-	+	+	-	-	+	+	-	+	+	-	-	+	+	-	3+	2+	4+
2	R_1R_1	+	+	-	-	+	-	+	+	-	+	/	+	+	-	-	+	-	-	+	+	-	+	+	-	+	+	-	-	-	2+
3	R_2R_2	-	+	+	+	-	-	+	+	-	+	/	+	+	+	-	+	+	-	+	-	+	+	+	-	+	-	-	3+	1+	4+
4	R_0	-	+	-	+	+	-	-	+	-	+	/	/	-	+	+	+	+	-	+	+	+	+	+	-	+	/	-	1+	1+	4+
5	r'r'	+	-	-	-	+	-	-	+	-	+	/	+	+	-	+	-	+	-	+	+	-	+	+	-	+	-	-	-	-	2+
6	r''r''	-	-	+	+	-	-	-	+	-	+	/	+	+	+	+	+	-	+	+	+	-	+	+	-	+	+	-	-	2+	2+
7	rr	-	-	-	+	+	-	+	-	-	+	/	+	+	+	+	+	-	+	+	+	-	-	+	-	+	+	-	-	1+	2+
8	rr	-	-	-	+	+	-	-	+	-	+	/	+	-	+	-	+	+	-	-	-	+	+	+	-	+	/	-	2+	2+	4+
9	rr	-	-	-	+	+	-	-	+	-	+	/	+	-	+	+	+	-	+	+	+	-	+	+	+	-	-	-	-	2+	2+
10	rr	-	-	-	+	+	-	-	+	-	+	/	+	+	-	-	+	-	+	+	-	+	+	-	-	+	/	-	3+	-	4+
11	R_ZR_1	+	+	+	-	+	-	-	+	-	+	/	+	-	+	+	+	-	+	-	+	+	-	+	-	+	+	-	1+	3+	4+
12	R_ZR_2	W	+	+	+	-	-	-	+	-	+	/	+	-	+	+	+	+	-	+	-	+	+	+	-	+	+	-	1+	3+	4+
13	r'^wr	+	-	-	+	+	+	+	+	-	+	-	+	+	-	+	-	-	+	-	+	-	+	+	-	+	+	-	-	-	2+
14	rr	-	+	+	+	-	-	-	+	-	+	/	+	+	-	+	-	-	+	+	+	-	-	+	-	+	+	-	-	-	2+
15	R_2R_2	+	-	-	+	+	-	-	+	-	+	/	+	+	+	+	-	+	-	+	+	+	-	+	-	+	-	-	2+	1+	4+
16	r'r	-	-	-	+	+	-	-	+	-	+	-	+	-	+	+	+	w	+	-	+	-	+	+	-	+	-	-	-	2+	2+

Note: +: indicates agglutinate reaction; -: no agglutinate and hemolysis reaction; /: indicates not involved.

Identification of Drug Antibody Subtypes

Using the immune-complex method, patient serum samples collected at different time points were serially diluted. Fifty μL of 1% standard group-O RBC suspension and 50 μL of diluted patient serum were added to a test tube, followed by 50 μL of 1 mg/mL drug solution. Normal pooled AB serum was used simultaneously as a negative control. The mixture was incubated at 37 °C for 15 min, centrifuged, and the results were recorded.

Table 6 Dynamic changes in vancomycin antibody titers and drug concentration

POD	Titer																	Ctl.
	IgM										IgG							
	1:1	1:2	1:4	1:8	1:16	1:32	1:64	1:128	1:256	1:512	1:2	1:4	1:8	1:16	1:32	1:64	1:128	
8	/	/	/	/	/	/	/	/	/	-	4+	3+	3+	3+	3+	2+	-	-
9	/	/	/	/	/	/	/	/	/	-	4+	3+	3+	2+	1+	1+	-	-
13	4+	4+	4+	4+	4+	4+	4+	3+	3+	-	4+	3+	3+	2+	2+	1+	-	-
69	4+	3+	3+	2+	1+	-	-	-	-	-	4+	3+	2+	1+	-	-	-	-
111	3+	2+	1+	-	-	-	-	-	-	-	1+	-	-	-	-	-	-	-
123	-	-	-	-	-	-	-	-	-	-	-	-	-	-	-	-	-	-

Note: 1. +: indicates agglutinate reaction; -: no agglutinate and hemolysis reaction; /: indicates not involved, mf: mixed field.

2. The numbers in "Days" column indicate the day after the surgery, the number "0" indicates the day on surgery.
